# Identifying opportunities to prevent work-related fatal injury in New Zealand using 40 years of coronial records: protocol for a retrospective case review study

**DOI:** 10.1186/s40621-019-0193-z

**Published:** 2019-05-06

**Authors:** Rebbecca Lilley, Bronwen McNoe, Gabrielle Davie, Simon Horsburgh, Brett Maclennan, Tim Driscoll

**Affiliations:** 1 Department of Preventive and Social Medicine, Dunedin School of Medicine, University of Otago, PO Box 56, Dunedin, 9054 New Zealand; 2 Public Health, School of Public Health, University of Sydney, Sydney, NSW Australia

**Keywords:** Occupational safety policy, Evaluation, Occupational safety, Injury, Fatal, New Zealand, Work-related, Road, Workplace

## Abstract

**Background:**

Improving New Zealand’s poor workplace safety record has become a high priority following high profile workplace fatal incidents in 2010 and 2014. Existing routine official data are unable to reliably inform occupational safety policy and action in New Zealand. This case review study will examine work-related fatal injury (WRFI) to: i) determine their burden, rates and distribution; ii) identify high-risk groups, causes and circumstances; iii) analyse secular trends, and iv) examine the impact of historic occupational safety legislative reform.

**Design and methods:**

A comprehensive New Zealand WRFI dataset from 1975 to 2014 will be established using existing data for 1975–1994 combined with new data for 1995–2014 extracted from reviewed coronial case files. Data collection involves: 1) identifying likely cases of WRFI from national mortality records using selected injury external cause codes; 2) linking these to coronial case files, which will be retrieved and reviewed to determine work-relatedness; and 3) coding work-related cases. Annual WRFI frequencies and rates will be calculated and disaggregated by age, sex, employment status, occupation and industry to identify high-risk groups and compared across the time series. The circumstances of the WRFIs will be analysed in-depth. The impact of New Zealand’s Health and Safety in Employment 1992 Act, which resulted in deregulation of the previous legislative frameworks for occupational health and safety during a period of rapid labour market restructuring, will be examined by comparing rates before and after implementation of the Act.

**Discussion:**

The resulting evidence will serve as the basis for policy development and practical interventions to reduce WRFI, targeting groups of high-risk workers, and for bench-marking of workplace safety performance in New Zealand.

## Background

Work-related injury is responsible for major health loss, disability and death in New Zealand (NZ) resulting in significant burdens and costs for individuals, workplaces and society. Every year one in ten workers is injured at work in NZ (Pearce et al. 2004; Independent Taskforce on Workforce Health and Safety 2013). The most recent estimates from 2004 indicate from a employed working population of 1.7 million over 100,000 workers require time off work, 12,000 are permanently disabled and 105 are killed every year from these injuries (Pearce et al. 2004). The community impact is wider still, with a further 115 work-related bystander deaths a year as a result of another person’s work activity (Langley et al. 2006). Inequalities in work-related injuries exist: the indigenous population of Māori workers is 1.6 times more likely to be killed at work (McCracken et al. 2001), and the social and economic impacts, estimated at $15–21 billion per annum (2–4% NZ GDP) (Access Economics 2006; Department of Labour 2011), are greatest for vulnerable low income, young families (Butcher 2004).

NZ’s workplace fatality record is very poor compared to similar countries, being twice that of Australia and four times that of the United Kingdom (Feyer et al. 2001a; Lilley et al. 2013). The reasons for NZ’s poor performance, including the performance of the occupational safety regulatory environment, are highly debated and inadequately informed due to a lack of high quality work-related fatality data (Independent Taskforce on Workforce Health and Safety 2013). The 29 fatalities at the 2010 Pike River mine explosion and the 2013/14 spike in Forestry work fatalities have placed NZ’s poor workplace safety record under close public scrutiny. The recent 2013 Independent Taskforce on Workplace Safety was, “strongly of the view that all workplace injuries are preventable, any death is unacceptable” (Independent Taskforce on Workforce Health and Safety 2013).

## Poor data to inform action

Currently in NZ, at the population level, it is not possible to accurately describe: i) who is fatally injured due to work, ii) what groups should be prioritised for action, and iii) what impact workplace safety regulation and interventions have had on preventing work-related fatalities. The most comprehensive and accurate estimates were produced in 2004 as part of a one-off research project. NZ’s official WRFI indicators exclude work-related road traffic and bystander fatalities and rely on linkage with NZ’s national injury treatment, rehabilitation and compensation provider Accident Compensation Corporation (ACC) work-related injury claims data which are heavily influenced by ACC claims management policy (Statistics New Zealand 2014). By focusing solely on workers, NZ’s official WRFI indicators miss important opportunities to reduce the broader public health impact of work-related fatalities. Disturbingly, these official routine data sources do not accurately capture or identify the total number of workers fatally injured, leading to a 40–60% underestimation of work-related fatal injuries in NZ (Langley et al. 2000). Inadequate data have been repeatedly highlighted as a serious impediment to reducing workplace injury in New Zealand (Pearce et al. 2004; Independent Taskforce on Workforce Health and Safety 2013; Langley et al. 2000; Pearce et al. 2006). The 2013 Taskforce that concluded poor quality data contributed to recent occupational safety system failings through “insufficient information and intelligence on risks, causes and interventions” (Independent Taskforce on Workforce Health and Safety 2013). The Taskforce identified as a priority the need for “more robust research and data” to inform prevention (Independent Taskforce on Workforce Health and Safety 2013).

## The work-related fatal injury study data series

The Work-Related Fatal Injury Studies-1 and -2 (WRFIS-1 & -2) created the most complete and detailed evidence platform for occupational safety policy and action in NZ by using a cohort study design of retrospective case reviews of coronial files to identify work-related fatalities (Cryer and Fleming 1987; Feyer et al. 2001b). Coronial files are records of Coronial Inquests undertaken as a legal requirement in response to sudden, unexpected fatality events, providing rich information on the decedent and the circumstances surrounding the injury event. A Coronial case file is theoretically available for all legally notified deaths, regardless of the location of death, with deaths occurring in hospital also notifiable to the Coronial system. The role of the Coroner, a legal representative, is to determine the cause and circumstances of a death and identify any lessons that can be drawn to prevent similar future deaths. While it is not the role of a Coroner to determine work-relatedness the completeness and detail rich nature of Coronial case files make them the “gold standard” in terms of capturing work-related cases compared with other official sources of data based on compensation or notification data.

WRFIS-1 & -2 (covering the period 1975–94) identified many new areas of workplace safety concern (Pearce et al. 2004; Langley et al. 2006; Feyer et al. 2001b; McNoe et al. 2005) and in-depth examination of high-risk groups revealed opportunities to intervene and control common causes of fatal injury. Furthermore, WRFIS-2 identified how official New Zealand data can misrepresent prevention priorities by: a) underestimating workplace deaths by up to 60%; and b) excluding work-related road traffic and bystander deaths thereby missing up to a further 60% of the total 221 annual work-related fatalities (Langley et al. 2006; Feyer et al. 2001b). The WRFIS-1 & -2 data are now over twenty years old, and cannot provide an indication of current trends relating to today’s mix of workers, occupations and industries that have shifted substantially in that time (Gander et al. 2009). Updated accurate data on the current patterns of work-related fatalities are urgently needed as it remains unknown how the safety performance of historically high-risk groups, such as older workers, males and workers in high hazard industries like forestry and logging, have improved, stagnated, or declined since 1994 or how the causes of injury may have changed.

The addition of data to 2014 will add value to WRFIS-1 & -2 by providing the only continuous national coronial dataset to establish long-term trends (over a 40 period of 1975–2014) and to assess the impact of changes in the distribution of the workforce and of previous legislative reform on work-related fatalities.

## Aim and objectives

The aim of this research is to accurately inform directions for work-related fatal injury prevention efforts. Through epidemiological analyses of new (1995–2014) and existing (1975–1994) high quality, information-rich coronial data with comprehensive capture of work-related fatalities, the specific objectives are to: i) determine the total burden, rates and distribution of WRFI; ii) identify the current and long-term high-risk groups, causes and circumstances to prioritise and target action; iii) analyse secular trends in WRFI and iv) examine the impact of New Zealand’s major 1992 occupational safety legislative reform on WRFI. A secondary function of this project will be to assess the feasibility of using available electronic coronial case files for regular, timely and efficient future surveillance of work-related fatalities.

## Design and methods

This research project aims to address the current deficit in knowledge about WRFI by using coronial case file data to establish a complete and comprehensive cohort of all work-related fatal injuries from 1975 to 2014. The project consists of two parts; the first, establishing the coronial dataset and the second, analysing and interpreting the data. Ethical approval for this study was granted by the University of Otago Human Ethics Committee (Ref 15/065), National Coronial Information System (Ref NZ007), and Health and Disability Ethics Committee (Ref OTA/99/02/008/AM05).

## Part 1: establishing the coronial-based WRFI dataset

**Fig. 1:**
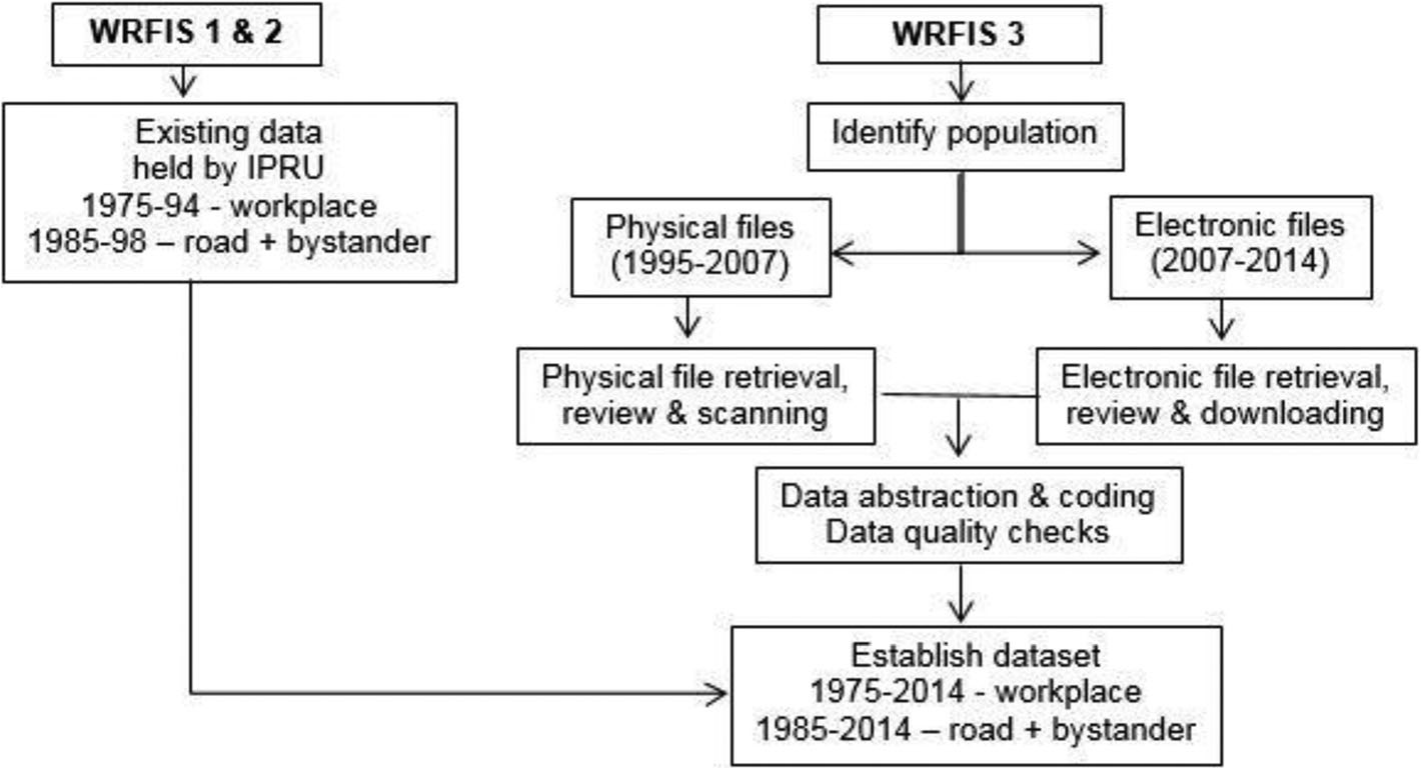
Flow diagram of methodological processes to establish the coronial-based WRFI dataset

The research team possesses the data from WRFIS-1 & -2. The valid and feasible methods used in WRFIS-1 & -2 (Cryer and Fleming 1987; Feyer et al. 2001b; McNoe et al. 2005) will be replicated to collect data from work-related injury fatalities occurring during 1995–2014 (referred to hereafter as WRFIS-3), ensuring consistency across the three WRFIS projects. The methodological schema for establishing the 40-year dataset is presented in Fig. 1.

### Procedure for constructing WRFIS-3 dataset

#### Definitions of work-relatedness and classification of cases

A broad definition of work-relatedness, compared with official data definitions, will be used to allow maximal utility and comparability with national and international data; we intend to capture all fatalities to which a workplace exposure contributed. The work-relatedness of a fatal injury event will be decided based on whether the decedent, at the time of the fatal incident, was: working for pay, profit or payment in kind; assisting with work in an unpaid capacity; was engaged in other work-related activities even when on a break or away from the workplace; or was a bystander to another person’s work activity (Cryer and Fleming 1987; Feyer et al. 2001b). Road-traffic work-related fatal injuries occurring to both workers and bystanders on public roads will be included to capture the entire population of workplace fatal injuries (road-traffic fatalities were excluded from WRFIS-1) (Langley et al. 2006; McNoe et al. 2005). The specific case inclusions & exclusions are listed in Table 1, and are consistent across historic and new data collection periods.

**Table 1:** WRFIS-3 study inclusion and exclusion criteria

Included fatalities	Excluded fatalities
The primary inclusion criteria are fatal injury of people working for pay, profit or payment-in-kind (such as unpaid family working with a family business) Specific circumstances from this primary category that will be eligible include fatal injuries: • occurring away from the workplace or in a non-work period, but to which work contributed; • to the foreign crew of boats, ships or airplanes, if the injury occurs on NZ soil or within NZ coastal waters or air space; • involving trains and traffic on public or private roads; • of persons who are helping in the family business; • of self-employed persons, as well as employees; and • of those assisting with work activities in unpaid capacity as official volunteers or students. • of persons who are New Zealand or non-New Zealand citizens. The secondary inclusion criteria is fatal injury of bystanders to someone else’s work activity, such as to members of the public.	Individuals less than 18 years of age, or greater than 85 years of age at time of death. Fatal injury of persons performing unpaid home duties. Deaths due to occupational diseases. Deaths due to domestic violence. Specific injury types that will be excluded include deaths due to legal intervention, medical misadventure or complications, suicide and self-inflicted injury, or operations of war.

All fatal injury cases determined to be work-related will be broadly classified as follows:

- **Working deaths:** fatal incident occurred in the course of work duties (referred to as workplace WRFI), including deaths involving road-traffic on a public road (referred to as road-traffic WRFI).
- **Bystander deaths:** the decedent was not working but died as a result of someone else’s work activity regardless of fault (referred to as bystander WRFI).

The use of a broad classification of WRFI will allow the use of differing categorisations and definitions of work-related injury fatalities during analysis, thus allowing for increased data utility.

#### Identify population of injury fatalities

Potential WRFI cases with a date of death on or between 1/1/1995 and 31/12/2014 will be identified using New Zealand’s Mortality Collection, the most complete data source for all New Zealand deaths, including work-related injury fatalities. The Ministry of Health’s (MoH) Mortality Collection classifies the underlying cause of death (UCoD) for all deaths registered in NZ using the International Classification of Diseases (ICD). For deaths due to injury and poisoning, the cause of death is classified using External Cause codes (E-codes). As in WRFIS-1 & -2, injury deaths will be identified as any death in the Mortality Collection with an UCoD classified to the following ICD 9th Revision E-codes: E800-E869, E880-E928 or E950-E999. Since fatalities from 1/1/2000 are classified using ICD 10th revision, ICD mapping files from MoH will be used to maintain consistency. To reduce the number of cases needing manual review we will use “selected contributing causes”, available in the Mortality Collection since 1999, to further identify injury fatalities that meet the exclusion criteria. Based on currently available data and the exclusions listed above, there are around 1250 potentially relevant injury deaths per year, and thus, approximately 25,000 records that will require review for the period 1995–2014.

#### Linkage to coronial case files

Once the population of injury fatalities has been identified, data on the decedents will be linked to the corresponding coronial case file. As there is no common unique identifier in both the Mortality Collection and coronial files, cases will be linked using surname, first given name, second given name, age, date of birth, and date of death. Wherever possible, we propose to use surnames and first names for matching based on results from previous WRFIS-2 data matching and previous published studies that demonstrate links between hospital and police road crash records without name returned 50% linkage, whereas names resulted in 90% linkage (Feyer et al. 2001b). In WRFIS-2, using the described linkage process, coronial case files were identified for 95% of the injury deaths identified from the Mortality Collection (Feyer et al. 2001b).

#### Additional methods of case selection

Other sources of fatal injury data will be used to help identify further potential WRFI for review, including specific occupational and road traffic injury administrative or notification data collections; mainly Accident Compensation Corporation (ACC), New Zealand Transport Authority (NZTA) and WorkSafe NZ. Previous WRFI studies have identified small numbers of new cases (10 cases for 1985–94) using these three sources, indicating comprehensive coverage of work-related fatalities by the Mortality Collection (Langley et al. 2006). Although the number of additional cases identified by these data sources is likely to be small (< 10), their continued use in WRFIS-3 allows us to create the most complete set of work-related injury fatality data possible.

#### Data collection: coronial extraction and review to determine work-relatednes

The methods for coronial case file review closely follow those employed by previous WRFIS (Cryer and Fleming 1987; Feyer et al. 2001b; McNoe et al. 2005). All potential cases will be reviewed using event descriptions available from coronial case files and classified according to the study case definitions, as “definitely work-related”, “definitely not work-related” or “indeterminate”. Deaths classified as “work-related” or “indeterminate” will have all relevant available material (such as Coroner’s findings, and witness, police, pathology and toxicology reports) extracted and securely transferred to the research team’s permanent repository of work-related files in Dunedin. All “indeterminate” coronial case files will be reviewed by the Dunedin-based research team and a final determination of work-relatedness made. Physical case files will be obtained from the Coronial Services of NZ, Wellington, while electronic files will be obtained from the National Coronial Information System (NCIS), Australia.

#### Data collection: coding

Confirmation of the work-relatedness of each file will be undertaken by the Dunedin-based coder immediately prior to coding. The demographic information on the decedent, and information about their work and workplace, the fatal incident and the type of injuries sustained will be extracted from coronial files. The standard coding frameworks used in WRFIS-1 and -2 will form the basis for data coding, ensuring consistency across all WRFIS and to allow future international comparisons to be made. The main coding frames are presented in Table 2.

**Table 2:** Main Coding frames to be used in WRFIS-3

Variable(s)	Coding frame used
Usual place of residence, Socio-economic deprivation (NZdep)	Mortality Collection domicile codes (Ministry of Health 2014)
Physiological cause of death, mechanism of injury, agent of incident & injury, location of injury	Type of Occurrence Classification System (National Occupational Health and Safety Commission 1990)
Location of incident	National Data Standards for Injury Surveillance Place of Incident (National Injury Surveillance Unit 1995)
Occupation, at time of injury & usual	NZ Standard Classification of Occupations 1999 (Statistics New Zealand 2001)
Industry, at time of injury & usual	Australian & NZ Standard Industrial Classification, 1996 (Statistics New Zealand 1999)

Additional variables, consistent with previous WRFIS, to be collected include employment type, temporal factors (e.g. time of day, day of week), vehicle/road factors (e.g. vehicle type), and contributing factors (e.g. alcohol, drug). The coronial case files, which commonly include pathology, police and toxicology reports, will provide the main source of information to be coded, consistent across historic and new data collection periods. With the electronic files, coded data from NCIS are also available alongside the raw coronial case files. Extra coded information on vehicle and environmental factors will be available from NZTA files for road-traffic deaths.

#### Data collection: data quality & reliability

Similar to WRFIS-2, regular quality-control meetings and processes will ensure the production of high quality reliable data and allow for concordant decision-making and coding across datasets. Both inter- and intra-rater agreement for coding will be assessed. A random sample of 100 coronial files identified from the Mortality Collection as injury fatalities will be independently reviewed for determination of work-relatedness by all case reviewers, both at the beginning and the end of the review process. Intra-rater reliability will be determined by comparing the determination of work-relatedness between the beginning and end of the review process. Inter-rater reliability will be determined by comparing between reviewers. The reasons behind any inconsistencies between reviewers in determining work-relatedness will be examined further. Based on our experience with the WRFIS-2, we expect coding reliability to be high in trained coders, with better than 0.9 Kappa agreement in most cases.

Consistency in coding will be determined using a random sample of 50 files coded independently by all members of the coding team two months into the twelve-month coding process. The level of percentage agreement between key variables will be determined. The reasons behind any inconsistencies between coders will be examined further and if necessary files will be re-coded. Based on our experience with previous WRFIS, we expect 90–100% agreement on most key variables. At the conclusion of coding there will be a comprehensive review of all variables looking for out-of-range values or illogical values or combinations of values.

#### Calculation of incidence rates

An individual’s average risk of WRFI will be assessed using rates per 100,000 workers. To calculate incidence rates for 1995–2014, annual Census-derived estimated resident working population counts by year, age, gender and prioritised ethnicity will be obtained from Statistics NZ. Worker denominators from the 1996, 2001, 2006, and 2013 Censuses by year, age, gender, prioritised ethnicity, employment status, occupation and industry will also be obtained from Statistics NZ with denominators for inter-censal years estimated by linear interpolation. We will also calculate rates per 100,000 full-time equivalent (FTE) workers using denominator data that takes account of part-time workers’ reduced exposure. Estimates from the Household Labour Force Survey will be used as denominators for FTE worker rates.

### Procedure to create the 40-year dataset (1975–2014)

Data from WRFIS-3 will be appended to data from WRFIS-1 & -2 to create the 40-year dataset (1975–2014). Comparative coding of a subset of 50 records selected at random from the first two studies will be undertaken to assess percentage agreement in ascertainment and classification of work-relatedness for WRFIS-1 & -2 with WRFIS-3. The same will be undertaken for some key variables (i.e.. mechanism of injury) allowing for an assessment of the consistency in case ascertainment and coding across the three studies.

## Part 2: data analysis and interpretation

In the following “current” refers to the most recent 10 years of data (2005–2014) and “historical” refers to the 40 years of data available for fatal injuries that occurred to workers in the workplace (1975–2014) and the 30 years of data available for workers in road-traffic and for bystanders (1985–2014).

As the 2011 Christchurch earthquake will have an impact on the number of WRFI in 2011, we will present for comparison, where appropriate, analyses that exclude injuries due to natural disasters.

### Determine the total burden, rates and distribution of WRFI

Using the historical dataset, annual frequencies with 95% Confidence Intervals (CIs) calculated assuming Poisson error for WRFI overall, and separately by work-related activity (i.e. workplace, road-traffic and bystander WRFI). Annual incidence rates per 100,000 workers will be presented with exact Poisson CIs. The presence of a linear increase or decrease in rates over each 10 year-period and the full 40-year period will be assessed using Poisson regression.

The contribution of WRFI to all fatal injuries will be calculated for each year. The proportion of injury deaths classified as WRFI and the corresponding 95% exact binomial CIs will be presented using the historical time-series. Change over time will be assessed using regression methods. This approach will be extended with the proportion of WRFI to injury deaths calculated by gender, age group and external cause.

### Identify high risk groups and patterns of risk over time

For workers only, work-related injury fatalities will be aggregated by 10-year periods with incidence rates of WRFI with 95% CIs calculated by age group, gender, prioritised ethnicity, employment status, occupation and industry to identify groups with high rates of WRFI. Linear change over the four decades and comparisons of trends between groups will be analysed using Poisson regression using all available data points. Historical aggregated WRFIS data by work-related activity indicate we will have sufficient statistical power for these analyses. Possible explanations for differences in observed rates over-time will be examined by using direct standardisation to account for changes in age, sex, ethnicity, occupational and industry distributions. Relative Risks (RR) and 95% CIs calculated using crude and standardised risk estimates will be compared to evaluate the impact of changes in workforce composition over-time upon WRFI.

Following an approach used in the United States, it is proposed that groups with a rate twice the overall mortality rate will be considered “high-risk” (Minnesota Department of Health 2015). The percentage of workers employed in occupations and industries classified as at high risk of occupational mortality using this approach will be calculated to provide an indication of the level of occupational/industrial exposure to high-risk in the working population.

### Identify common causes and circumstances

The person, work, time (e.g. time of injury), environment (e.g. incident location), injury (e.g. cause of death), incident (e.g. mechanism and agent of incident) and contributing factors (e.g. drugs, alcohol) will be described using both frequencies and proportions with corresponding 95% CIs for workers. The distribution of causes and circumstances of WRFI occurring within occupation and industry groups will also be examined using tests for proportions and chi-squared tests as appropriate. Changes over time will be assessed using chi-squared tests for trends and regression models. For the subset of road-traffic worker deaths, frequencies of WRFIs by vehicle type (e.g. heavy truck, van) and the proportion that have recorded drugs and/or alcohol involvement will be calculated.

### Identify areas of emerging high risk of WRFI

Using historical data on workers, separate Poisson regression models will be used to estimate trends in the rates of WRFI by age, sex, ethnicity, occupation and industry. Areas/groups for which the trend line has a large positive slope will be identified as having emerging risk. Population projections obtained from Statistics NZ will be combined with extrapolated rates predicted from the regression models and used to forecast the likely number of WRFI that will be observed up to 2025 if rates do not change.

### Examine the impact of 1992 occupational safety legislative reform

The Health and Safety in Employment (HSE) 1992 Act, enacted in 1993, signalled a major change in the approach to preventing occupational injury in NZ by replacing many ad-hoc, sector-specific, prescriptive items of legislation with a single act based on performance-based duties of care with voluntary industry standards across most places of work (Independent Taskforce on Workforce Health and Safety 2013). An implicit objective of this Act was a reduction in the rate of WRFI and this study will test whether this occurred, overall and by subgroup (i.e. worker, bystander), by comparing WRFI rates from before the HSE 1992 Act (1975–1992) with those after (1993–2014).

Since bystander WRFI and road-traffic WRFI are included in the HSE 1992 Act, analyses including all WRFIs will be conducted using 30 years of data from WRFIS-2 and WRFIS-3. Since this timespan includes a limited number of years from which to estimate the trend before the Act, fatality data will be aggregated with WRFI rates calculated using four time periods (pre: 1985–1992, post1: 1993–1999, post2: 2000–2006, post3: 2007–2014). Multivariable Poisson regression will be used to model the three pre-post changes. Using available aggregated denominator data, the model will be extended to adjust for changes in the worker populations by age, gender, prioritised ethnicity and employment status over time.

## Part 3: assessing the feasibility of future electronic surveillance

A secondary function of this project is to assess the feasibility of using electronic coronial case files available via the NCIS from 2007 for regular, timely and efficient surveillance of work-related fatalities. Electronic records provide additional pre-coded summary data alongside Coroner’s findings, toxicology, pathology and police reports, but exclude witness statements and other agency reports such as WorkSafe NZ reports. The completeness and validity of using a single existing coded data field within electronic files for identifying either worker or bystander fatalities will be assessed by comparing key data fields, such as the Coroner-determined work-relatedness field or case type field in the electronic record with the WRFIS-3 determination of work-relatedness for the period 2007–14. The level of agreement between the electronic record variable and the WRFIS-3 determination of work-related fatalities will be calculated for single data fields and for combinations of these using our study data for the period 2007–2014. This will enable the best combination of NCIS data fields for capturing cases of WRFI to be identified for ongoing electronic surveillance purposes.

## Discussion

This research will produce high quality, accurate data covering a 40-year period. To our knowledge, this will be the first study to cover WRFIs in New Zealand and internationally within a 40-year time period. The resulting research evidence will make an important contribution towards the development of workplace safety policy and practical interventions to reduce WRFI and provide benchmarks for workplace safety performance comparisons, data identified as an urgent priority by New Zealand’s Independent Taskforce on Workplace Health and Safety (Independent Taskforce on Workforce Health and Safety 2013). Our research findings will directly inform Government agencies with responsibilities for workplace health and safety of short- and long-term work-related fatal injury trends, and of groups of workers and circumstances with higher risk of fatal injury. WRFIS-1 & -2 have provided the most comprehensive examination to date of the nature and circumstances of fatal injuries in the New Zealand workplace (Pearce et al. 2004). This project will extend our previous research by delivering new comprehensive high quality information on the current and long-term trends on all New Zealand WRFI, groups of workers at risk of fatal injury and the circumstances involved, crucial for accurately identifying targets for policy and interventions for prevention and allowing for international benchmarking of safety performance (Feyer et al. 2001a; Driscoll et al. 2004). It will also be the first time the impact of the 1992 deregulation of occupational safety on trends in fatalities is examined using robust data, informing debate over the performance of this legislation and further establishing baseline data to evaluate the impact of impending occupational safety legislative reforms in New Zealand. The 40-year dataset will serve as a future resource for further interrogation to inform prevention activities to reduce work-related fatalities and serious non-fatal injury with similar exposures.

## Conclusions

This will be the first study to cover WRFIs in New Zealand and internationally within a 40-year time period. The resulting rigorous 40-year dataset will serve as a future resource for further interrogation to inform prevention activities to reduce work-related fatalities and serious non-fatal injury with similar exposures in New Zealand and to evaluate the performance of New Zealand’s workplace safety system.

## Abbreviations

ACC: Accident Compensation Corporation
CI: Confidence Interval
E-code: External cause code
GDP: Gross Domestic Product
HSE Act: Health and Safety in Employment Act
ICD: International Classification of Diseases
MoH: Ministry of Health
NCIS: National Coronial Information System
NZ: New Zealand
NZTA: New Zealand Transport Authority
RR: Relative risk
UCoD: Underlying cause of death
WRFI: Work-related fatal injury
WRFIS: Work-related fatal injury study
